# Chinese Herbal Medicine (Yiqi-Yangyin-Jiedu Decoction) Combined With Osimertinib as First-Line Treatment in EGFR Mutation-Positive Advanced Non-Small-Cell Lung Cancer (CATLA-2): A Study Protocol for a Double-Blind Randomized Controlled Trial

**DOI:** 10.3389/fphar.2022.840889

**Published:** 2022-04-01

**Authors:** Jialin Yao, Yan Lu, Lijing Jiao, Ling Bi, Wenxiao Yang, Lingzi Su, Jun Shi, Zhe Wang, Yabin Gong, Ling Xu

**Affiliations:** ^1^ Department of Oncology, Yueyang Hospital of Integrated Traditional Chinese and Western Medicine, Shanghai University of Traditional Chinese Medicine, Shanghai, China; ^2^ Institute of Clinical Immunology, Yueyang Hospital of Integrated Traditional Chinese and Western Medicine, Shanghai University of Traditional Chinese Medicine, Shanghai, China

**Keywords:** non-small cell lung cancer, epidermal growth factor receptor, osimertinib, Yiqi-Yangyin-Jiedu decoction, Chinese medicine

## Abstract

**Background:** Epidermal growth factor receptor (EGFR) tyrosine kinase inhibitors (EGFR-TKIs) significantly improve the prognosis of non-small cell lung cancer (NSCLC) with EGFR mutation-positive. Although third-generation EGFR-TKI osimertinib is demonstrated with superior efficacy compared with first-generation EGFR-TKIs, acquired resistance to EGFR-TKIs remains the bottleneck. The Chinese herbal medicine (CHM) Yiqi-Yangyin-Jiedu decoction (YYJD) has been shown to delay acquired resistance to first-generation EGFR-TKIs in the CATLA study, but there is no high-level evidence for its effect when combined with osimertinib. This trial aims to evaluate the efficacy and safety of YYJD combined with osimertinib as first-line treatment in EGFR mutation-positive advanced NSCLC.

**Methods:** This is a double-blind, multi-center, randomized controlled trial conducted in eight hospitals in China. A total of 314 participants will be randomly assigned to the osimertinib plus YYJD group (O+YYJD) or the osimertinib plus placebo group (O+placebo). Treatment will last until disease progression or death. Patients diagnosed with advanced NSCLC harboring EGFR Ex19del or L858R will be enrolled if they are ready to take osimertinib as first-line treatment, aged 18–74 years old, and provide signed informed consent. The primary outcome is progression-free survival (PFS). The secondary outcomes include a comparison of overall survival (OS), objective response rate (ORR), disease control rate (DCR), and quality of life (QoL). The analysis will be based on intention-to-treat and per-protocol subject analysis principles.

**Discussion:** The goal of this trial is to evaluate the efficacy and safety of YYJD when added to osimertinib as first-line treatment in EGFR mutation-positive advanced NSCLC.

**Trial Registration:** Chictr.org.cn, identifier ChiCTR1900026748

## Introduction

Non-small cell lung cancer (NSCLC) is the leading cause of cancer death worldwide (Siegel et al., 2020). Epidermal growth factor receptor (EGFR) is the most common driver gene for NSCLC, and approximately 40% of NSCLC patients in Asian populations have EGFR mutations (Gelatti et al., 2019). The most common mutation types in EGFR include exon 19 deletion (Ex19del) and exon 21 Leu858Arg (L858R), often referred to as “classical” mutations (Gazdar, 2009), which account for about 85% of EGFR mutations (Harrison et al., 2020). In addition, 12%–34% of NSCLC patients with EGFR mutations have uncommon mutations (Yang et al., 2015; Castellanos et al., 2017; Robichaux et al., 2018), which include exon 20 insertion, exon 18 G791X, exon 20 S768I, and exon 21 L861Q mutations. However, evidence on the efficacy of treatment for patients with uncommon mutations is insufficient. First-generation epidermal growth factor receptor tyrosine kinase inhibitors (EGFR-TKIs), such as gefitinib (Fukuoka et al., 2011) and erlotinib (Rosell et al., 2012), significantly improve the prognosis of advanced NSCLC patients harboring EGFR-TKI-sensitizing mutations, mainly EGFR Ex19del and L858R, when compared with traditional platinum-based chemotherapy as first-line treatment. Despite initial responses, most patients develop acquired resistance to first-generation EGFR-TKIs, and the disease will progress within 9–11 months (Fukuoka et al., 2011; Rosell et al., 2012). Third-generation EGFR-TKIs such as osimertinib have been shown to delay disease progression and prolong survival time compared to gefitinib (Soria et al., 2018; Ramalingam et al., 2020). Despite this, acquired resistance to osimertinib develops over a median of 18.9 months, so optimizing the effect of osimertinib is critical to the long-term survival of NSCLC patients.

Chinese herbal medicine (CHM) has been used in Chinese NSCLC patients treated with EGFR-TKIs for more than 10 years and has demonstrated its efficacy in delaying EGFR-TKI resistance and alleviating adverse effects in a number of clinical trials (Yang et al., 2018; Jiao et al., 2019; Tang et al., 2019). CHM contains several active compounds that interact with target proteins involved in EGFR-TKI resistance (Bing et al., 2018). Our previous multi-center, double-blind, placebo-controlled clinical trial (CATLA study) confirmed that the addition of CHM (Yiqi-Yangyin-Jiedu decoction, YYJD) to EGFR-TKI (gefitinib, erlotinib, or icotinib) significantly prolongs progression-free survival (PFS) and improves the quality of life (QoL) in NSCLC patients (Jiao et al., 2019). These trials mainly involve first-generation EGFR-TKIs. Osimertinib, as a third-generation EGFR-TKI, inhibits EGFR-sensitive mutations (Ex19del and L858R) like the first-generation EGFR-TKI. Given their similar mechanisms of action, it is reasonable to expect CHM to have clinical benefits for osimertinib as well.

The current study (CATLA-2) will determine whether the addition of CHM YYJD to third-generation EGFR-TKI osimertinib (O+YYJD) prolongs PFS compared with osimertinib plus placebo (O+placebo) in advanced NSCLC patients who have an activating EGFR mutation.

## Methods and Analysis

### Study Design

**FIGURE 1:**
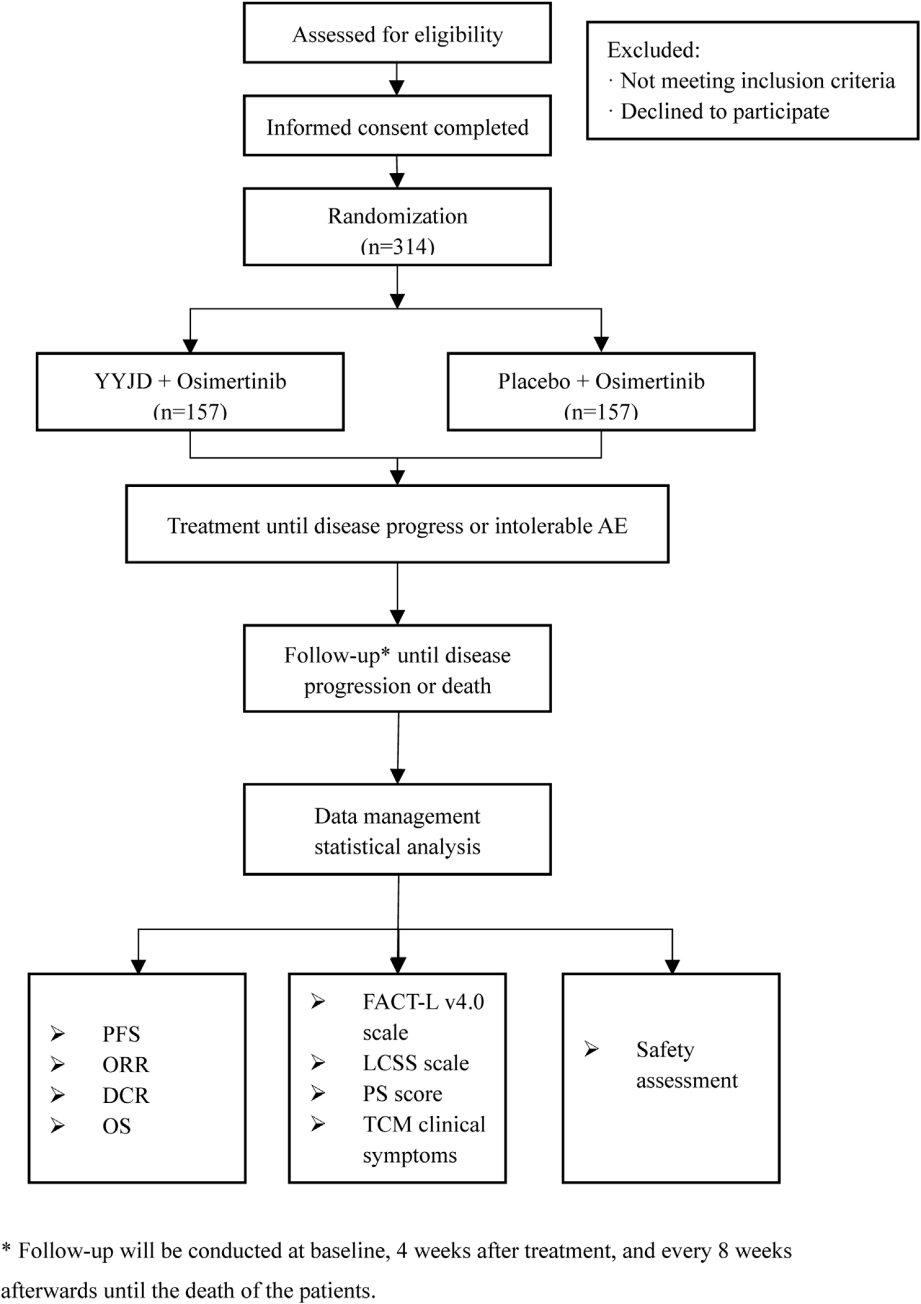
The flow diagram of the study.

This is a multi-center, double-blind, randomized controlled trial, which will be conducted in Yueyang Hospital of Integrated Traditional Chinese and Western Medicine Affiliated to Shanghai University of Traditional Chinese Medicine, Shanghai Pulmonary Hospital Affiliated to Tongji University, Shanghai Chest Hospital Affiliated to Shanghai Jiaotong University, Guang’anmen Hospital of China Academy of Chinese Medical Sciences, First Affiliated Hospital of Guangzhou University of Traditional Chinese Medicine, First Affiliated Hospital of Tianjin University of Traditional Chinese Medicine, Affiliated Hospital of Chengdu University of Traditional Chinese Medicine, and Jiangsu Provincial Hospital of Traditional Chinese Medicine. The study aims to enroll 314 advanced NSCLC patients with EGFR mutation-positive. Patients will be randomized at a ratio of 1:1 to receive either osimertinib plus YYJD or osimertinib plus placebo. Follow-up will be conducted at baseline, 4 weeks after treatment, and every 8 weeks afterward until disease progression or death, which is evaluated according to Response Evaluation Criteria in Solid Tumors (RECIST) version 1.1. The study design is based on the Standard Protocol Items: Recommendations for Interventional Trials (SPIRIT) 2013 statement (Chan et al., 2013). The flow diagram of the study is shown in Figure 1.

### Participants

Participants will be recruited through the outpatient and inpatient wards of the eight sites. Posters and digital media will be used for recruitment.

#### Inclusion Criteria

(1) Pathologically confirmed NSCLC (Health Commission of PRC and National Health Commission of the People’s Republic of China, 2019).
(2) Locally advanced or metastatic NSCLC that is not amenable to curative surgery or radiotherapy.
(3) The tumor has one of 2 common EGFR mutations known to be associated with EGFR-TKI sensitivity (Ex19del, L858R).
(4) Patients must have no treatment for locally advanced or metastatic NSCLC.
(5) Aged 18–74 years old.
(6) No major organ dysfunction: hemoglobin ≥120 g/L, absolute neutrophil count (ANC) ≥1.5 × 10^9^/L, platelets ≥80 × 10^9^/L, bilirubin ≤ 1.5ULN, alkaline phosphatase (AP), aspartate aminotransferase (AST), and alanine aminotransferase (ALT) ≤ 2.5 × ULN. INR ≤ 1.5, creatinine ≤ 1.5 × ULN.
(7) Provision of informed consent forms.

#### Exclusion Criteria

(1) Those with a history of other malignancies within 5 years;
(2) Those with symptomatic brain metastases;
(3) Those with congestive heart failure (> class II NYHA heart function), unstable (angina at rest), initial onset (starting within 3 months), angina pectoris, or myocardial infarction occurring within 6 months;
(4) Those with active infection (> Grade 2 adverse events according to CTCAE.5.0 version);
(5) Those with a history of uncontrollable mental illness.

#### Drop-Out Criteria

(1) Those who do not comply during the trial have an impact on the evaluation of effectiveness and safety;
(2) Those who withdraw themselves during the trial;
(3) Those who combine drugs, especially drugs that have a large effect on test drugs, affect effectiveness and safety evaluation;
(4) Those who withdraw from the trial, do not receive follow-up, or die before the end of the treatment course for other reasons;
(5) Those with incomplete information that affects the evaluation of effectiveness and safety;

The investigators will continue to evaluate the safety after they have been dropped out.

#### Voluntary Withdrawal of Participants

Participants are allowed to withdraw from the trial at any time, according to the informed consent form. Participants who do not formally withdraw from the trial but stop receiving intervention treatment or testing on their own, or are lost to follow-up, are considered to have withdrawn. The reasons for all participants who withdraw from the trial should be identified and documented to the extent possible.

### Sample Size

The sample size was calculated by the statistician of the Clinical Evaluation Center of Shanghai University of Traditional Chinese Medicine by the Power Analysis and Sample Size (PASS) 14 software. PFS is the primary endpoint. According to our previous study, the median PFS of the control group is expected to be 18 months and that of the intervention group is expected to be 23 months, with a recruitment duration of 18 months and a total duration of 30 months. The two groups were allocated in a ratio of 1:1. Given that *α* = 0.05, *β* = 0.20, considering that the average monthly censored rate is 1%, the total sample size is 314 cases, 157 cases in each group.

### Randomization

Eligible patients enrolled at each site will be randomly allocated to either the O+YYJD group or the O+placebo group at a 1:1 ratio through a dynamic random method. When patient gender (male vs. female), age (≥65 years old vs. <65 years old), and enrollment center is input as stratified factors, the software will automatically output the results of randomization. Personnel for drug administration will be able to obtain a random number and group allocation immediately in the form of a short message service and complete drug distribution according to the allocation.

### Blinding

The randomization results and blinding codes will be kept strictly confidential. They will be concealed until interventions are all assigned and enrollment, follow-up, data collection, data cleaning, and analysis are completed. Participants and researchers, including paramedics, investigators, outcomes assessors, and statisticians, will be unaware of the allocation.

### Interventions

Patients in the O+YYJD group will receive osimertinib and YYJD granule. Patients in the O+placebo group will receive osimertinib and placebo.

#### Yiqi-Yangyin-Jiedu Decoction

YYJD is produced into granules by Tianjiang Pharmaceutical Co., Ltd. (Jiangyin, Jiangsu Province, China) as previously reported (Wang et al., 2018) and the composition of YYJD is shown in Table 1. YYJD granules are taken with 150 ml of warm water twice a day after breakfast and lunch.

**TABLE 1:** Composition of Yiqi-Yangyin-Jiedu decoction.

Drug name	Produced from	Dosage (g)
*Astragalus mongholicus* Bunge [Fabaceae]	Dry rhizoma	30
*Codonopsis pilosula* (Franch.) Nannf. [Campanulaceae]	Dry rhizoma	9
*Atractylodes macrocephala* Koidz. [Asteraceae]	Dry rhizoma	12
*Poria cocos* (Schw.) Wolf. [Polyporaceae]	Dry sclerotia	15
*Epimedium brevicornu* Maxim. [Berberidaceae]	Herbal	15
*Trigonella foenum-graecum* L. [Fabaceae]	Dry seed	15
*Cullen corylifolium* (L.) Medik. [Fabaceae]	Fruit	12
*Adenophora stricta* Miq. [Campanulaceae]	Dry rhizoma	30
*Glehnia littoralis* (A.Gray) F.Schmidt ex Miq. [Apiaceae]	Dry rhizoma	30
*Asparagus cochinchinensis* (Lour.) Merr. [Asparagaceae]	Dry rhizoma	15
*Ophiopogon japonicus* (Thunb.) Ker Gawl. [Asparagaceae]	Dry rhizoma	15
*Lilium brownii* var. *viridulum* Baker [Liliaceae]	Scale leaf	15
*Ligustrum lucidum* W.T.Aiton [Oleaceae]	Fruit	12
*Prunella vulgaris* L. [Lamiaceae]	Dry spikes	7.5
*Arisaema heterophyllum* Blume [Araceae]	Dry rhizoma	15
*Amorphophallus konjac* K.Koch [Araceae]	Dry tubers	15
*Cremastra appendiculata* (D.Don) Makino [Orchidaceae]	Dry pseudobulb	7.5
*Euphorbia helioscopia* L. [Euphorbiaceae]	Herbal	7.5
*Selaginella doederleinii* Hieron. [Selaginellaceae]	Herbal	15
*Salvia chinensis* Benth. [Lamiaceae]	Herbal	15
*Paris polyphylla* var. *chinensis* (Franch.) H.Hara [Melanthiaceae]	Dry rhizoma	7.5
*Ziziphus jujuba* Mill. [Rhamnaceae]	Fruit	4.5

#### Placebo

Placebo is produced into granules by Tianjiang Pharmaceutical Co., Ltd. (Jiangyin, Jiangsu Province, China) with the most similar package, color, smell, and shape to YYJD but without medical ingredients. It is taken with 150 ml of warm water twice a day after breakfast and lunch.

#### Osimertinib

Osimertinib (TAGRISSO, AstraZeneca, United Kingdom) should be taken at 80 mg per day. Patients enrolled in this study should continue intervention until disease progression or intolerable adverse effects.

### Outcomes

Computed tomography (CT) or magnetic resonance (MR) imaging will be used to assess the tumor at baseline, 4 weeks after treatment, and every 8 weeks afterward until disease progression or death. Objective response is evaluated according to RECIST 1.1, established by the National Cancer Institute (NCI), and divided into the following four situations: Complete Response (CR), Partial Response (PR), Stable Disease (SD), and Progressive Disease (PD).

#### Primary Outcome

PFS: measured with the date of the videography from a random assignment to the date of objective progression or death by the researcher.

#### Secondary Outcomes

(1) Overall survival (OS): calculated as the time from randomization to death due to any cause. For subjects who are lost to follow-up before death, the time of the last follow-up is used as the time of no death.
(2) Objective response rate (ORR): calculated based on the effective rate of CR+PR patients.
(3) Disease control rate (DCR): calculated based on the effective rate of CR+PR+SD patients.
(4) Physical condition: assessed following the ECOG PS standard at the same time point of image evaluation.
(5) QoL: evaluated with Functional Assessment of Cancer Therapy-Lung (FACT-L) questionnaire, Lung Cancer Symptom Scale (LCSS) and TCM syndrome score at the same time point of image evaluation. TCM syndrome score will be evaluated based on the grading scales of lung cancer symptoms required in the Guiding Principles of Clinical Research of New Chinese Medicine Treating Primary Bronchial Lung Cancer (2002 Edition) issued by the National Medical Products Administration of China.
(6) Safety assessments: Participants will be asked and all adverse events (AEs) during treatment will be recorded at each visit, and all AEs reported will be analyzed. Blood, urine, and stool routine, liver function, and kidney function are tested for adverse reactions at the same time point of image evaluation and evaluated according to common terminology criteria for adverse events (CTCAE) version 5.0 (https://ctep.cancer.gov).

### Participant Timeline

**FIGURE 2:**
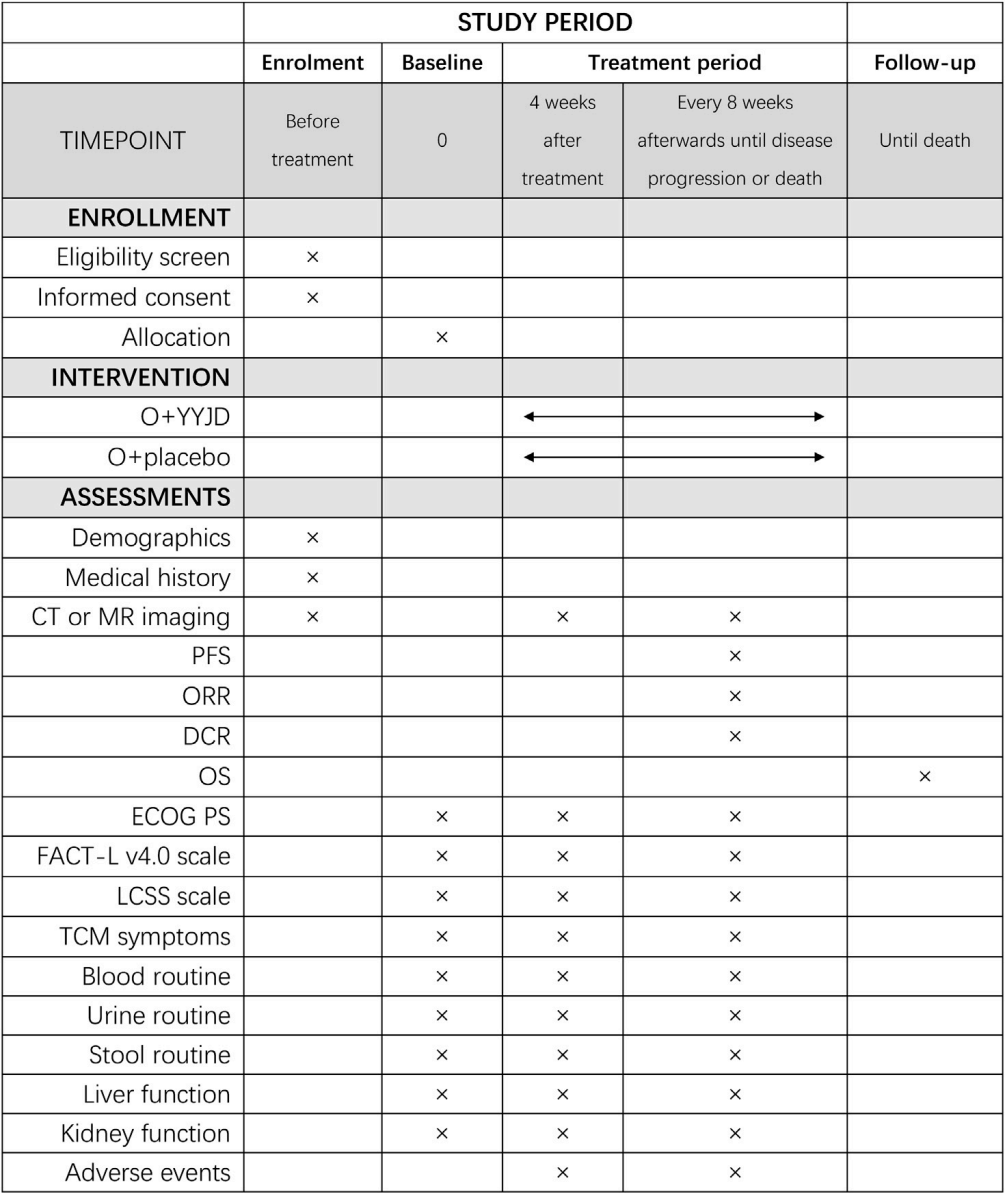
The schedule of enrolment, interventions, and assessments.

The schedule of enrolment, interventions, and assessments is as shown in Figure 2.

### Data Collection and Management

The data will be collected with the use of paper case report forms (CRF) by researchers from each center. Participants will be given a separate space to fill out the QoL form for privacy concerns. The data management specialist of each center will use the electronic data capture (EDC) system for data entry and management. The system will verify the entered data, and the questionable data will be confirmed or corrected by researchers after re-checking. The researchers responsible for data analysis can access the data only after the patient recruitment is completed. The sub-center will conduct a self-inspection every month, the main center will conduct an inspection of the sub-center every 3 months, and the competent unit of the study will conduct a project verification once every 6 months. Relevant researchers will need to reply and rectify any problems found within 2 weeks.

### Quality Control

All researchers will receive unified training so that they have a full understanding of clinical trials before the start of the trial. In order to improve patients’ compliance with osimertinib, we will provide detailed information on the benefits of using osimertinib as first-line treatment compared with first-generation EGFR-TKIs and inform patients that the first-line treatment of osimertinib for advanced non-small cell lung cancer with EGFR mutation-positive (Ex19del or L858R) can be reimbursed by medical insurance in China. This makes the financial burden caused by osimertinib relatively small and valuable.

### Statistical Analysis

Data analysis will follow the trial’s statistical analysis plan. All data will be processed by statistical analysis with SAS 9.4 software analysis. Two-tailed *p* values <0.05 are considered statistically significant. The analysis will follow intention-to-treat, full analysis set, and per-protocol subject principles for the evaluation of primary and secondary outcomes. The safety data set that includes all subjects who received at least one treatment after randomization will be used to evaluate safety and tolerance. Missing data will be processed with the multiple imputation method.

The baseline characteristics will be reported according to treatment groups. PFS and OS will be illustrated by the Kaplan–Meier survival curve and compared between groups using the log-rank test. The secondary outcomes will be summarized with frequency, mean, standard deviation, median, and range. At each time point, comparisons between the experimental group and the placebo group will be conducted using the Group t-test or Wilcoxon rank-sum test (for measurement data) and the rank-sum test and CMH test (for count data). Fisher’s exact test will be used to compare tumor response rates between the arms. For PFS, an adjusted Cox regression model will be used to estimate the adjusted HRs for differences between the treatment arms with the selected prognostic factors, including the center, EGFR mutation type, age, sex, EGFR-TKI drugs, smoking status, and ECOG PS.

Safety will be documented in adverse event forms and presented with descriptive statistics for each group. The frequency difference of adverse events between groups will be assessed by the chi-square test or Fisher’s exact test. For different AE severities, a rank-sum test will be performed to analyze the independent ordered multiple category data between the two groups.

### Dissemination Plans

The results will be published in a paper after the completion of the study.

### Trial Status

Protocol version: v2.0 was finished on 27th July 2020. Participant recruitment started in January 2021 and is expected to end in December 2022. Until 23rd December 2021, a total of 58 participants were enrolled in the study.

## Discussion

EGFR-TKIs are the standard first-line treatment for patients with locally advanced or metastatic EGFR-mutated NSCLC (Hanna et al., 2017; Planchard et al., 2018; Wu et al., 2019). Although the emergence of EGFR-TKIs has brought a revolutionary breakthrough in the treatment of lung cancer, the disease still progresses after 9–11 months of treatment with EGFR-TKIs (Fukuoka et al., 2011; Rosell et al., 2012). Patients with EGFR-mutated NSCLC who are initially sensitive to EGFR-TKI therapy will inevitably experience treatment failure due to secondary drug resistance, and more than 50% of them have the T790M mutation (John et al., 2018; Wang et al., 2018). The emergence of the third-generation EGFR-TKI, osimertinib, has solved this problem. The FLAURA study shows that the median PFS of patients treated with osimertinib is as high as 18 months, and its first-line treatment significantly prolongs OS to 38.6 months (Soria et al., 2018; Ramalingam et al., 2020). Therefore, osimertinib is the first choice for first-line treatment in EGFR mutation-positive advanced NSCLC. However, resistance and toxicities of osimertinib have become new challenges. Osimertinib resistance mechanisms include EGFR C797S mutation and MET gene amplification (Kim et al., 2015; Planchard et al., 2015). The toxic and side effects of osimertinib significantly affect the quality of life of patients with NSCLC and even lead to the cessation of treatment (Khozin et al., 2017).

As a complementary and alternative medicine, TCM is widely used in cancer treatment in China, which has shown the effects of reducing chemotherapy toxicity, enhancing tumor treatment response, improving quality of life, and prolonging survival (Zhang et al., 2018). In the CATLA study, we found that the combination of EGFR-TKIs (gefitinib, erlotinib, or icotinib) and CHM YYJD as first- and second-line therapy significantly prolonged PFS and ORR versus EGFR-TKIs alone. The median PFS was prolonged by 5 months as first-line treatment. At the same time, the side effects of EGFR-TKIs, including fatigue, loss of appetite, diarrhea, pruritus, and skin rash, were reduced (Jiao et al., 2019). Nowadays, the advantage of the third-generation EGFR-TKI, osimertinib, over the first-generation EGFR-TKI is that it overcomes the T790M resistance mutation and has better clinical efficacy in the treatment of advanced NSCLC patients with EGFR mutation-positive. It is reasonable to speculate that YYJD may also have the effect of reducing toxicity and increasing the efficiency of osimertinib.

Qi and Yin deficiency syndrome is one of the most common deficiency syndromes in TCM, manifesting as a pathological state in which both qi and yin deficiency coexist. The typical symptoms are cough, less sputum, fatigue, shortness of breath, dry mouth with less drinking, spontaneous sweating, reddish tongue, or tongue with teeth imprints, thready and weak pulse (Zheng, 2002). Meanwhile, the types of TCM syndromes under EGFR mutations in lung adenocarcinoma are dominated by Qi and Yin deficiency syndrome, and treatment should pay attention to tonifying Qi and nourishing Yin (Wang and Xu, 2017). YYJD is a CHM compound based on TCM theory that mainly exerts the effects of tonifying Qi, nourishing Yin, and detoxifying. In the subgroup analysis of the CATLA study, we found that YYJD had a better clinical benefit for advanced NSCLC patients with EGFR-sensitive mutations, extending median PFS by 16.26 months (Jiao et al., 2019). In addition, most trials have shown that the combination of EGFR-TKIs with oral CHM that tonifies Qi and/or nourishes Yin significantly delays acquired resistance while increasing the ORR of EGFR-TKIs (Lu et al., 2021). Therefore, in the design of this study, the intervention was set as YYJD with greater clinical benefit in order to minimize the impact of TCM syndrome differentiation on outcomes.

In summary, this study is a large-sample multicenter randomized controlled trial of osimertinib combined with YYJD as first-line treatment in EGFR mutation-positive advanced NSCLC. If effective, it will provide a high-quality evidence-based basis for the efficacy and safety of osimertinib combined with YYJD and provide a treatment strategy integrating Chinese and Western medicine that can be widely and successfully applied in the clinic.
